# Tailoring Iridium Valence States on ZSM-5 for Enhanced Catalytic Performance in CO Selective Catalytic Reduction of NO under Oxygen-Enriched Environments

**DOI:** 10.3390/ma17061440

**Published:** 2024-03-21

**Authors:** Yarong Bai, Chuhan Miao, Weilong Ouyang, Lang Wang, Haiqiang Wang, Zhongbiao Wu

**Affiliations:** 1 Key Laboratory of Environment Remediation and Ecological Health, Ministry of Education, College of Environmental Resources Science, Zhejiang University, Hangzhou 310058, China; byr987333@163.com (Y.B.); miaoch1018@163.com (C.M.); oywl123666@163.com (W.O.); 22114046@zju.edu.cn (L.W.); 2 Zhejiang Provincial Engineering Research Center of Industrial Boiler Furnace Flue Gas Pollution Control, Hangzhou 310058, China

**Keywords:** nitrogen oxides, selective catalytic reduction, carbon monoxide, oxygen-enriched, iridium, cyclic stability

## Abstract

Barium and iridium supported on Zeolite Socony Mobil-5 (ZSM-5) are efficient catalysts for the selective catalytic reduction of nitric oxide by carbon monoxide (CO-SCR), with enhanced cyclic stability. The introduction of Ba hindered the oxidation of metallic Ir active species and enabled Ir to maintain an active metallic state, thereby preventing a decrease in catalytic activity in the CO-SCR reaction. Moreover, the Ba modification increased the NO adsorption of the catalyst, further improving the catalytic activity. Owing to the better anti-oxidation ability of Ir^0^ in IrBa0.2/ZSM-5(27) than in Ir/ZSM-5(27), IrBa0.2/ZSM-5(27) showed better stability than Ir/ZSM-5(27). Considering that all samples in the present study were tested to simulate actual flue gases (such as sintering flue gas and coke oven flue gas), NH_3_ was introduced into the reaction system to serve as an extra reductant for NO*_x_*. The NO*_x_* conversion to N_2_ (77.1%) was substantially improved using the NH_3_-CO-SCR system. The proposed catalysts and reaction systems are promising alternatives for treating flue gas, which contains considerable amounts of NO*_x_* and CO in oxygen-enriched environments.

## 1. Introduction

In recent decades, anthropogenic atmospheric pollutants emitted during fossil fuel combustion have become a global issue in the context of climate change and air pollution. After 86% of the coal power in China reached the ultra-low emission policy targets by 2019, the focus shifted to other industrial sources such as steel plants, biomass-fired generators, and waste incinerators. Flue gases from these units typically contain considerable amounts of nitric oxide (NO) and high concentrations of carbon monoxide (CO), which can be considered a natural reductant for NO emissions [1,2,3].

The selective catalytic reduction of NO by CO (CO-SCR) is a promising method for controlling NO*_x_* emissions from stationary sources [4,5]. Previous studies on NO*_x_* reduction in vehicular emissions explored possible catalysts under O_2_-free or O_2_-deficient conditions [6,7,8,9,10]. Certain studies have reported that catalysts are active under O_2_-rich conditions but have been tested under low NO and high CO concentrations (CO:NO>100:1, only for automotive applications) [11,12] or using He as a balanced gas in the activity evaluation (inconsistent with actual applications) [13,14]. However, because CO is considerably more reactive with O_2_ than with NO*_x_*, it is challenging to use the original CO to reduce NO*_x_* when excess O_2_ exists in the flue gases [3,13,15,16,17,18]. It has been proven that Ir-based catalysts can achieve relatively high NO*_x_* conversion in the presence of excess O_2_ [19,20,21].

Ir loaded onto SiO_2_ catalysts can be used for CO-SCR reactions in the presence of O_2_, according to previous reports [20,22,23]. Molecular sieves are typical silicon-based materials with regular pore structures and have been widely used in many areas [24,25,26,27,28,29]. Therefore, we deduced that iridium loading onto molecular sieves (ZSM-5, SAPO-34, SBA-15, and MCM-41) may also obtain high NO*_x_* removal efficiency. Because Ir^0^ sites are the active sites for the CO-SCR reaction, better anti-oxidation of Ir-based catalysts in the presence of O_2_ is vital for the catalytic performance of these catalysts. Haneda et al. proved that Ba can inhibit the oxidization of Ir metal, which could be the active site for the CO-SCR reaction [21,30]. We deduced that Ba may also improve the CO-SCR performance of Ir-based molecular sieve catalysts.

In this study, an IrBa(0.2)/ZSM-5(27) catalyst was developed to obtain a bimetallic catalyst configuration with enhanced activity for NO*_x_* reduction by CO. Ba incorporation and H_2_ pretreatment at 500 °C improved the cyclic stability of the catalysts and N_2_ selectivity in the cyclic tests. The physicochemical properties of the Ba-Ir bimetallic catalyst were compared with those of the Ir monometallic catalyst to rationalize the functions of the Ba counterpart in the CO-SCR reaction.

## 2. Materials and Methods

### 2.1. Catalyst Preparation

All catalysts were prepared by impregnating zeolites with an aqueous solution of Ir and Ba precursors. The precursor of Ir used in this work is hexachloroiridium acid hydrate, while the precursor of Ba is barium nitrate. A total of 5 g of hexachloroiridium acid hydrate was dissolved in 200 mL of deionized water to form a solution in which the concentration of Ir was 0.008 g/mL. A total of 0.5 g barium nitrate was dissolved in 50 mL of deionized water to form a transparent solution. Both of these chemical reagents were purchased from Shanghai Aladdin Biochemical Technology Co., Ltd., Shanghai, China. H-ZSM-5 and SAPO-34 were purchased from Tianjin Nankai University Catalyst Co., Ltd., Tianjin, China. The Si/Al ratios of H-ZSM-5 and SAPO-34 were 27 and 0.5, respectively. Pure silica SBA-15 and MCM-41 molecular sieves were purchased from XFNANO Material Technology Co., Ltd., Nanjing, China.

In the preparation progress of Ir/ZSM-5 with a 0.5% Ir loading amount, 5 g of H-ZSM-5 was added to 50 mL of deionized water under stirring, and then 3.12 mL of hexachloroiridium acid solution (0.008 g/mL) was added to the above-mentioned solution. After stirring at room temperature for 6 h, the mixture was dried at 100 °C for 8 h. The sample, after drying, was calcined in an air atmosphere at 500 °C for 4 h, and the obtained powder was labeled as Ir/ZSM-5(27). The preparation method of Ir/SAPO-34, Ir/SBA-15, and Ir/MCM-41 was similar to that of Ir/ZSM-5(27); only the carrier of catalysts needed to change. Furthermore, Ir/ZSM-5 catalysts with other loading amounts were prepared using similar methods; only the addition amount of hexachloroiridium acid solution changed. The preparation method of Ba-modified Ir/ZSM-5(27) catalysts was the same as with Ir/ZSM-5(27); only barium nitrate solution needed to be added at the same time as hexachloroiridium acid was added to the solution. The molar ratio of Ba:Ir was set at 0.1, 0.2, 0.4 and 1.0. The prepared catalysts were labeled as IrBa0.1/ZSM-5, IrBa0.2/ZSM-5, IrBa0.4/ZSM-5, and IrBa1/ZSM-5, respectively.

### 2.2. CO-SCR Performance Evaluation

**Scheme 1:**
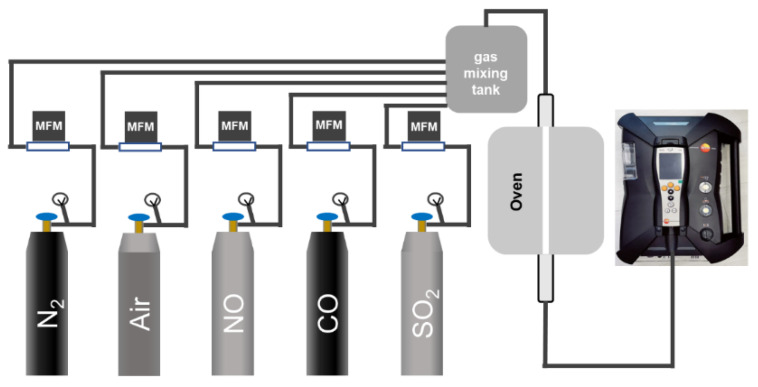
The catalytic performance testing equipment.

Selective catalytic reduction of NO with CO was carried out in a fixed-bed quartz reactor by using catalysts of 40–60 mesh (Scheme 1). The catalyst was placed in the middle position of the quartz reaction tube, and a thermocouple was placed above the catalyst to measure the reaction temperature during the activity test. The typical composition of the inlet gas was 600 ppm NO, 3000 ppm CO, 5 vol.% O_2_, and balanced with N_2_. A total of 2.3 mL of catalysts was used during the activity test, and the total gas flow was set at 1500 mL/min. The reaction temperature was tested from 180 °C to 500 °C; each reaction temperature was maintained for 30 min to reach a steady state. A total of 1.25 g of catalyst was used in each test, and the gas hourly space velocity (GHSV) corresponded to 40,000 h^−1^. The outlet concentrations of NO and NO_2_ were monitored by a flue gas analyzer (Testo 350, Testo Inc., Lenzkirch, Germany). The catalytic activity was evaluated in terms of NO conversion and NO*_x_* conversion, as follows:

$$\mathrm{NO}\;\mathrm{conversion}\;=\;\frac{\left[\mathrm{inlet}\;\mathrm{NO}\right]-\left[\mathrm{outlet}\;\mathrm{NO}\right]}{\left[\mathrm{inlet}\;\mathrm{NO}\right]}\times 100\%$$

$${\mathrm{NO}}_{x}\;\mathrm{conversion}=\frac{\left[\mathrm{inlet}\;{\mathrm{NO}}_{x}\right]-[\mathrm{outlet}\;{\mathrm{NO}}_{x}]}{\left[\mathrm{inlet}\;{\mathrm{NO}}_{x}\right]}\times 100\%$$

### 2.3. Catalyst Characterization

Transmission electron microscope (TEM) images were acquired on a FEI Tecnai G2 F20 S-TWIN (Hillsboro, OR, USA) instrument. The energy-dispersive X-ray spectroscopy elemental mapping (EDX-mapping) was acquired by an Oxford X-MAX 80T (Oxfordshire, UK) instrument. All images were acquired from the unreduced catalysts.

The crystal structures of the as-made catalysts were measured by powder X-ray diffraction (XRD) on an X-pert Powder (PANalytical B.V., Almelo, The Netherland). X-ray photoelectron spectroscopy (XPS, Thermo ESCALAB 250, Waltham, MA, USA) was used to investigate the surface properties of the samples with Al Kα radiation (*hν* = 1486.6 eV). The shift of the binding energy due to relative surface charging was corrected using the C 1s level at 284.8 eV as an internal standard. The specific surface areas were determined by the Brunauer–Emmett–Teller (BET) method on a nitrogen adsorption apparatus (ASAP 2020, Altanta, GA, USA). All the samples were degassed at 300 °C prior to measurements. The data were collected in relative pressure (P/P_0_) ranging from 0.05 to 0.30.

Temperature-programmed reduction of H_2_ (H_2_-TPR) was performed on a TP-5079 (Xianquan, Tianjin, China) using 50 mg samples. Prior to TPR experiments, catalysts were pretreated under a flow of 5 vol.% O_2_/He at 400 °C for 1 h and then cooled to room temperature. Reduction was carried out by heating the sample in 6 vol.% H_2_/N_2_ at a heating rate of 10 °C/min. The consumption of H_2_ was detected by a TCD detector. Temperature programmed desorption of NO (NO-TPD) was conducted on a similar TP-5079 setup. Catalysts were saturated with NO/He (1000 ppm NO) after pretreatment in He gas flow at 400 °C. Desorption was carried out by heating the sample in He gas flow from 70 °C to 800 °C (Hiden Analytical QGA, Warrington, UK).

## 3. Results and Discussion

### 3.1. The Influence of Catalyst Carrier and Ir Loading Amount on Catalytic Performance

**Figure 1:**
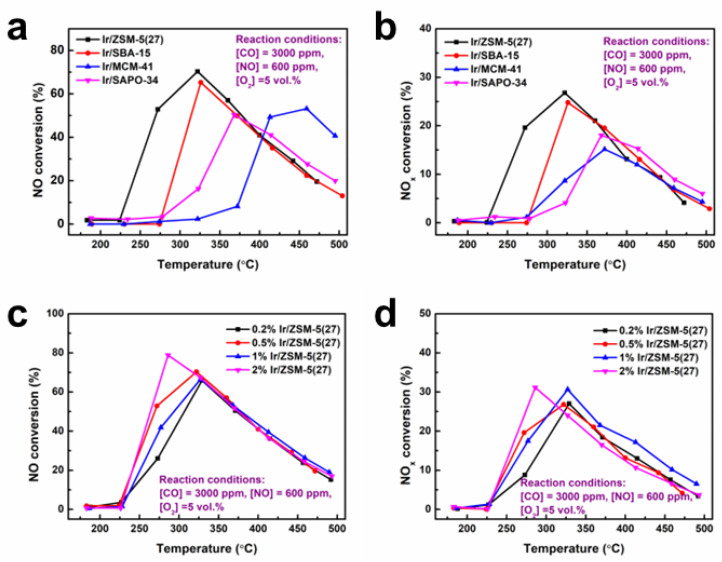
(**a**) NO and (**b**) NO*_x_* conversion on Ir-loaded zeolites without reduction. (**c**) NO and (**d**) NO*_x_* conversion on Ir/ZSM-5(27) with different Ir loading amounts.

The carrier plays a vital role in catalytic performance. Four types of zeolites were selected as typical supports for loading the Ir catalysts: ZSM-5 (Si/Al = 27), SAPO-34 (Si/Al = 0.5), SBA-15 (Si), and MCM-41 (Si). The NO and NO*_x_* conversions over the prepared catalysts are shown in Figure 1a,b. The catalysts used in this part were not pretreated with H_2_. Among the selected zeolite types, Ir/ZSM-5(27) exhibited the best NO and NO*_x_* conversion (70.3% and 26.8%, respectively) at 322 °C. The catalytic activity of Ir/SBA-15 was slightly lower than that of Ir/ZSM-5(27); 65.3% NO conversion and 24.8% NO*_x_* conversion were achieved using this catalyst. Moreover, the NO and NO*_x_* conversions of Ir/SAPO-34 and Ir/MCM-41 were lower than those of Ir/ZSM-5(27). A considerably better catalytic activity was obtained for Ir/ZSM-5(27) at low temperatures; thus, ZSM-5 was selected as the catalyst carrier for Ir in subsequent experiments.

Figure 1c,d shows the catalytic activity of unreduced Ir/ZSM-5(27) with different Ir loading amounts; the highest NO conversion was obtained when the Ir loading was increased to 2%. However, only 65.8% NO and 27.0% NO*_x_* conversions were obtained using 0.2% Ir/ZSM-5(27). Furthermore, the catalytic activity of 0.5% Ir/ZSM-5(27) at low temperatures was better than those of 0.2% Ir/ZSM-5(27) and 1% Ir/ZSM-5(27). Considering the high cost of Ir, the loading amount of Ir was set to 0.5% for subsequent investigations.

### 3.2. Optimization of Reduction Pretreatment

**Figure 2:**
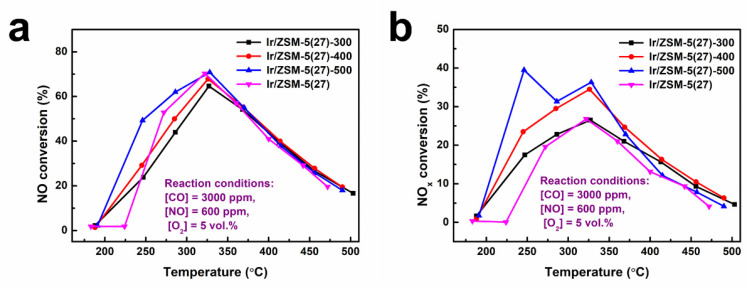
(**a**) NO and (**b**) NO*_x_* conversions on Ir/ZSM-5(27) that were treated at various reduction temperatures.

Ir-based catalysts become active in the CO-SCR reaction upon H_2_ pretreatment, which can improve the surface enrichment and ordering of Ir atoms [12,13]. Therefore, the effects of H_2_ pretreatment on the catalyst performance were studied using Ir/ZSM-5(27) samples with and without pretreatment in flowing H_2_/N_2_ gas, and their NO and NO*_x_* conversions are shown in Figure 2. The pretreatment with H_2_ at 500 °C improved the NO conversion of Ir/ZSM-5(27) in a temperature range of 190–280 °C. More importantly, NO*_x_* conversion by the catalyst improved substantially. The NO*_x_* conversion of the catalyst without H_2_ pretreatment was calculated as 0.1% at 224 °C. After pretreatment with H_2_/N_2_ gases at 500 °C, the NO*_x_* conversion of the catalyst increased to 39.5% and 36.3% at 246 °C and 328 °C, respectively. This change is attributed to the different Ir valence states in the catalysts, which were further explored using various characterization techniques, as described in the following section. Notably, the NO*_x_* conversion of the catalyst increases with increasing pretreatment temperatures. Furthermore, the NO*_x_* conversion was lower than the NO conversion owing to the transformation of NO to NO_2_, particularly in the temperature range from 270 °C to 400 °C.

Previous studies [12,21,31,32] have shown that Ir^0^ species, rather than their oxidized IrO_2_ form, are the active sites for NO reduction with CO. The highly dispersed Ir on the ZSM-5 surface was easily reduced during the pretreatment process, resulting in a considerable fraction of the catalytically active zero-valent Ir species being exposed to the small Ir particles, thereby enhancing the catalytic activity of the catalyst.

### 3.3. Influence of Ba Addition

**Figure 3:**
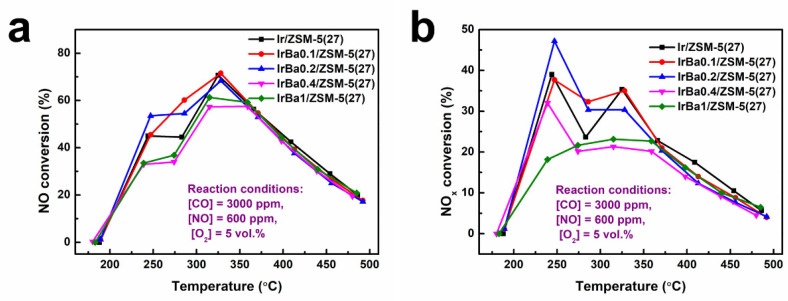
(**a**) NO and (**b**) NO*_x_* conversions of Ba modified Ir/ZSM-5(27) after reduction.

Previous reports have demonstrated that Ba could serve as a promoter to increase NO*_x_* conversion in the CO-SCR reaction because Ba could inhibit the oxidation of Ir^0^ sites [21,30,33,34]. Therefore, the influence of the Ba addition on the catalytic activity of Ir/ZSM-5(27) was investigated. Figure 3a shows the NO conversion of the Ba-modified Ir/ZSM-5(27) catalyst, where the NO conversion increased slightly at low temperatures (<300 °C) when the molar ratio of Ba to Ir was 0.1 and 0.2. However, the NO conversion decreased dramatically when the molar ratio of Ba to Ir was <0.2. The NO conversion did not change after the Ba modification. This result demonstrates that Ba modification mainly influences the catalytic activity at low temperatures (<300 °C) while having a negligible effect on the NO conversion of these catalysts at high temperatures (>300 °C). Figure 3b shows that 47.1% NO*_x_* conversion was obtained for IrBa0.2/ZSM-5(27) at 247 °C, which is obviously higher than that of Ir/ZSM-5(27) (only 39.0%). The above results show that Ba addition can improve catalytic activity at low temperatures but has little effect at high temperatures. It should also be noted that IrBa0.2/ZSM-5(27) showed a relatively higher NO conversion in the presence of O_2_ with a low Ir loading amount (Table 1).

### 3.4. The Stability of Catalysts and the Influence of Other Components on CO-SCR Catalytic Performance

**Figure 4:**
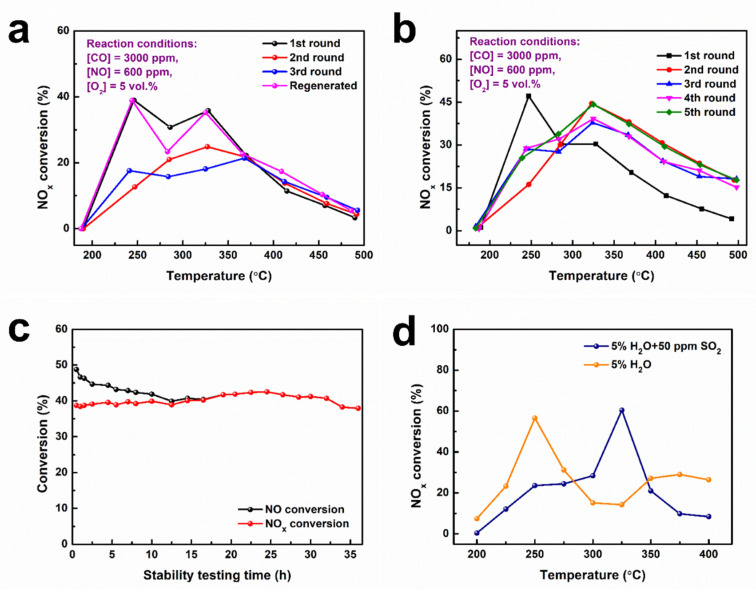
Cyclic performance and stability. (**a**) NO*_x_* conversion of Ir/ZSM-5(27), (**b**) NO*_x_* conversion of IrBa0.2/ZSM-5(27), (**c**) stability test of IrBa0.2/ZSM-5(27) at 320 °C, (**d**) the influence of H_2_O and SO_2_ on CO-SCR catalytic performance of IrBa0.2/ZSM-5. Feed composition: 600 ppm NO, 3000 ppm CO, 5 vol.% O_2_, 5 vol.% H_2_O (when used), 50 ppm SO_2_ (when used) balanced with N_2_, GHSV = 40,000 h^−1^.

Cyclic stability is vital for catalysts used in industrial applications. Therefore, the cycling performance of Ir/ZSM-5(27) was evaluated (results shown in Figure 4a). After three cyclic tests, the activity of the Ir/ZSM-5(27) sample significantly decreased in the temperature range of 190–370 °C, and the highest NO*_x_* conversion decreased from 39.0% (at 246 °C) to 21.4% (at 368 °C). The Ir/ZSM-5(27) catalyst exhibited poor cyclic stability, which requires further improvement before industrial application. After three cyclic tests, the deactivated Ir/ZSM-5(27) catalyst was then regenerated with H_2_, and the activity of the regenerated catalyst was basically recovered to its original activity, with NO conversions of 38.9% at 244 °C and 35.2% at 325 °C (that at 285 °C slightly decreased). These results suggest that the deactivation of the Ir catalysts was mainly caused by the loss of Ir^0^ (oxidized to IrO_2_) during the reaction, thereby diminishing the de-NO*_x_* ability after three cyclic tests and allowing easy recovery by H_2_ treatment.

As proposed, the addition of Ba hindered the oxidation of the active species, thus preventing a decrease in catalytic activity. Therefore, Ba was used as a promoter to improve the catalyst’s cycling performance. As shown in Figure 4b, NO*_x_* conversion at low temperatures (<280 °C) decreased for IrBa0.2/ZSM-5(27) after the first test. However, the NO*_x_* conversion of this catalyst at high temperatures (>280 °C) was increased after the first test, and nearly 40.0% NO*_x_* conversion (320–330 °C) was achieved during the cyclic performance test. The above results showed that the IrBa0.2/ZSM-5(27) catalyst could maintain more than 40.0% NO*_x_* conversion at approximately 320 °C after five times cycle use, indicating that Ba addition markedly improved the cyclic stability of the catalyst and prevented a decrease in catalytic activity.

A long-term stability test of IrBa0.2/ZSM-5(27) was performed at 320 °C for 36 h (Figure 4c). The NO*_x_* conversion was maintained above 38.0% throughout the test, demonstrating that this catalyst possesses robust resistance to O_2_ during the CO-SCR reaction. Long-term stability is vital for the practical use of catalysts. Furthermore, the effect of H_2_O on the CO-SCR activity of IrBa0.2/ZSM-5(27) was investigated (Figure 4d). Obviously, the NO*_x_* conversion at low temperatures increased after 5% H_2_O was introduced into the inlet gas, which is consistent with a previous report [36,37] because H_2_O can react with CO via a water–gas shift reaction to generate H_2_, which can be used to reduce the high-valence Ir species. However, NO*_x_* conversion decreased at low temperatures and increased at high temperatures after adding SO_2_. The inhibitory effect of SO_2_ on the catalytic performance at low temperatures is due to the presence of SO_2_, which can inhibit the conversion of CO, resulting in the ignition temperature of the CO-SCR reaction shifting to a high temperature [38].

### 3.5. NH_3_-CO-SCR Performance on the Ba-Modified Catalyst

**Figure 5:**
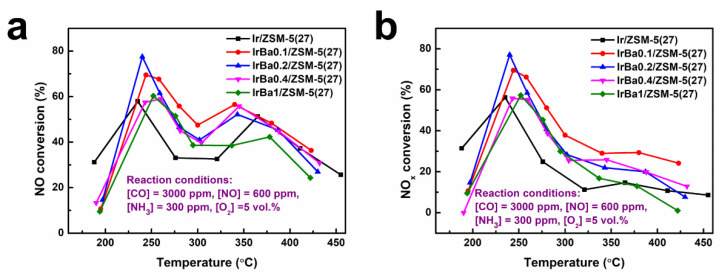
(**a**) NO and (**b**) NO*_x_* conversions on Ba-modified Ir/ZSM-5(27) catalyst during the NH_3_-CO-SCR reaction.

Although IrBa0.2/ZSM-5(27) achieved 47.1% NO*_x_* conversion in the presence of O_2_ (Figure 3b), this was far from the requirement for practical applications. Thus, 300 ppm NH_3_ was introduced into the reaction system. It was found that 77.1% NO*_x_* conversion (240 °C) was achieved on this catalyst (Figure 5b). This demonstrates that the addition of NH_3_ can significantly improve NO*_x_* conversion via the NH_3_-SCR process. Furthermore, the NO*_x_* conversion was close to the NO conversion at low temperatures (<280 °C) (Figure 5a,b), which was due to the addition of NH_3_ inhibiting the oxidization of NO to NO_2_ in the presence of excess O_2_. Notably, both NO and NO*_x_* conversions significantly improved after Ba modification, proving that Ba modification is an effective measure for improving the catalytic activity of Ir/ZSM-5(27).

### 3.6. Physical Properties of as-Made Catalysts

**Figure 6:**
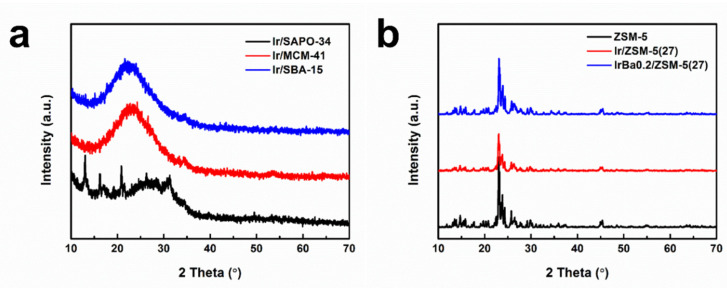
(**a**) The X-ray diffraction (XRD) patterns of Ir/SAPO-34, Ir/MCM-41 and Ir/SBA-15. (**b**) The X-ray diffraction (XRD) patterns of ZSM-5, Ir/ZSM-5(27) and IrBa0.2/ZSM-5(27).

As shown in Figure 6a, a broad peak around 20–30° appeared in the XRD patterns of Ir/SBA-15 and Ir/MCM-41, corresponding to the diffraction peak of amorphous silica. Notably, no peaks corresponding to Ir species were observed in the XRD patterns of Ir/SBA-15, Ir/MCM-41, and Ir/SAPO-34. Furthermore, the characteristic peaks of the MFI skeleton structure belonging to ZSM-5 were observed in the Ir/ZSM-5(27) and IrBa0.2/ZSM-5(27) spectra (Figure 6b), whereas the peaks of the Ir and Ba species were not observed in the IrBa0.2/ZSM-5(27) spectra [39,40,41]. This suggests that the Ir and Ba species were highly dispersed on the carrier, which could be attributed to the low loading of Ir and Ba during the preparation of this catalyst.

**Figure 7:**
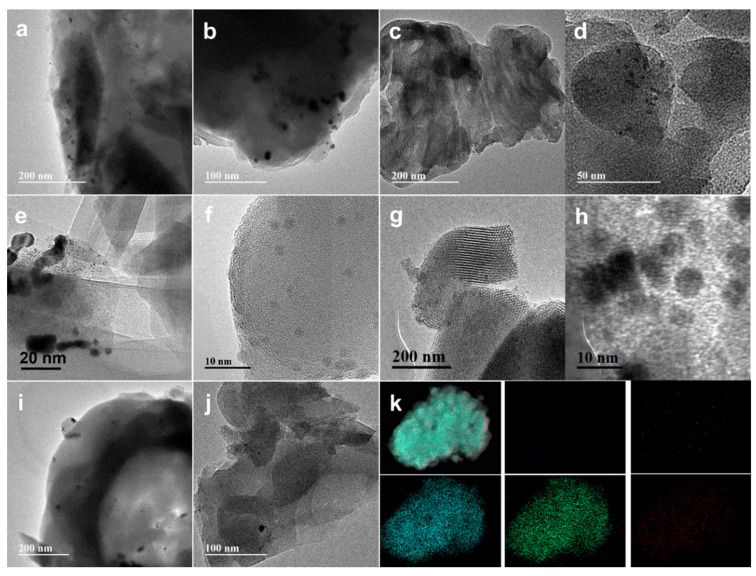
TEM images of Ir/ZSM-5(27) (**a**,**b**), Ir/MCM-41 (**c**,**d**), Ir/SAPO-34 (**e**,**f**), Ir/SBA-15 (**g**,**h**), and IrBa0.2/ZSM-5(27) (**i**,**j**). EDX-mapping of IrBa0.2/ZSM-5(27) (**k**).

Figure 7 shows the transmission electronic microscope (TEM) images of different samples. Because the relative molecular mass of Ir is heavier than that of Al and Si, the black dots in the TEM images correspond to the Ir species. As shown in Figure 7a,b, the Ir species in Ir/ZSM-5(27) mainly existed as large particles, and the diameters of most particles were close to 10 nm. However, the particle size of the Ir species in Ir/MCM-41 was only approximately 2 nm, which suggests the Ir species have better dispersion on MCM-41 than on ZSM-5 (Figure 7c,d). Moreover, a high dispersion of Ir species was also obtained in SAPO-34, and most of the Ir species existed in the form of small particles (Figure 7e,f). Furthermore, some large particles (>20 nm) were observed for Ir/SAPO-34 (Figure 7e). The Ir/SBA-15 catalyst exhibited regular mesoporous channels with a pore diameter of approximately 7 nm (Figure 7g). Some Ir nanoparticles were located in the channels of SBA-15, which is because these channels of SBA-15 are large enough to permit Ir species to enter the inner space of SBA-15. The particle size of the Ir species in Ir/SBA-15 was approximately 6 nm, as shown in Figure 7h. The particle size of the metal species decreased when Ba was also present in the catalyst, which means that Ba modification could decrease the agglomeration of Ir and result in better dispersion of the Ir species on ZSM-5 (Figure 7i,j). The different particle sizes of the Ir species on different carriers may result in different chemical properties of these catalysts, and Ba modification could also influence the chemical properties of Ir/ZSM-5(27). Energy-dispersive X-ray (EDX) spectroscopic elemental mapping was used to investigate the elemental distribution of IrBa0.2/ZSM-5(27) (Figure 7k). It is clear that both Ir and Ba were highly distributed on the surface of ZSM-5(27).

**Figure 8:**
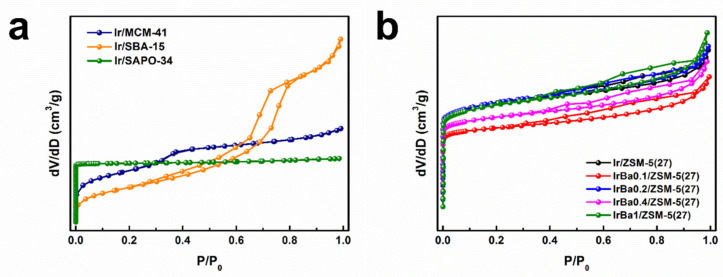
N_2_ adsorption/desorption isotherms of Ir loaded on different carriers (**a**) and Ba-modified Ir/ZSM-5(27) catalysts (**b**).

Ir/SBA-15 showed typical type IV isotherms with an H1 hysteresis loop, indicating the presence of mesoporous structures in the catalyst (Figure 8a). Ir/SAPO-34 shows typical I isotherms, demonstrating that this catalyst is a microporous material. Furthermore, Ir/ZSM-5(27) and Ba-modified Ir/ZSM-5(27) showed typical I isotherms (Figure 8b), which also suggests the existence of micropores in these catalysts.

The specific surface areas of Ir/SAPO-34 and Ir/MCM-41 were larger than that of Ir/ZSM-5(27), whereas the NO conversion of these two catalysts was worse than that of the Ir/ZSM-5(27) (Table 2). This result proved that the specific surface area of the catalyst had little effect on the catalytic performance of Ir-based catalysts. Furthermore, the specific surface area of ZSM-5 decreased after loading with Ir and Ba, possibly because some of these species entered the internal pores of ZSM-5. Although the BET surface area of IrBa0.2/ZSM-5(27) is smaller than that of Ir/ZSM-5, the catalytic activity of IrBa0.2/ZSM-5(27) was higher than that of Ir/ZSM-5. This result further demonstrates that there is no clear relationship between the specific surface area and the catalytic activity of these catalysts. Based on the above results, it is reasonable to deduce that the chemical properties (such as the NO adsorption ability and chemical valence of Ir) may play an important role in the catalytic performance.

### 3.7. Adsorption and Redox Properties of as-Made Catalysts

**Figure 9:**
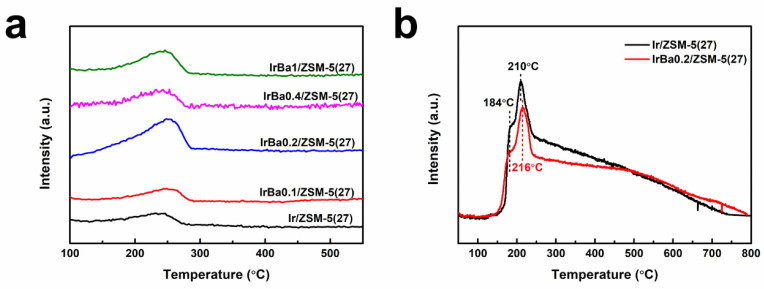
(**a**) NO-TPD and (**b**) H_2_-TPR spectrum of as-made catalysts.

Temperature-programmed desorption of NO (NO-TPD) tests were conducted on two samples to investigate the NO adsorption and desorption performances of Ir/ZSM-5(27) and IrBa0.2/ZSM-5(27). As shown in Figure 9a, the NO-TPD spectrum of Ir/ZSM-5(27) has a clear desorption peak between 100 °C and 300 °C (peaking at 244 °C), which might be due to weak adsorption species (nitrite and NO) because NO was the predominant species detected using mass spectrometry [42,43]. The Ba-modified catalysts’ desorption peaks became more pronounced, suggesting that Ba modification substantially increased the NO adsorption/desorption on Ir/ZSM-5(27). NO can adsorb on two different active sites of the Ir crystal. The NO adsorbed on the hollow sites was unstable and could further dissociate into N and O at low temperatures (even at room temperature), whereas the NO adsorbed on the atop sites was stable at low temperatures [44,45]. IrBa0.2/ZSM-5(27) and IrBa1.0/ZSM-5(27) may provide more atop sites, leading to greater desorption on these two catalysts than on IrBa0.4/ZSM-5(27). Moreover, the desorption peak area of IrBa0.2/ZSM-5(27) was markedly larger than the other samples, which may further improve its catalytic activity.

The H_2_ temperature-programmed reduction (H_2_-TPR) profiles of Ir/ZSM-5(27) and IrBa0.2/ZSM-5(27) were tested from 100 °C to 550 °C with a ramping rate of 10 °C/min. As shown in Figure 9b, the H_2_-TPR spectrum of Ir/ZSM-5(27) has a pronounced reduction peak of 210 °C with a shoulder at 184 °C, which can be attributed to the reduction of IrO_2_ to metallic Ir^0^ [15,25,26]. With Ba modification, the reduction peak at 210 °C shifted backward slightly to 216 °C. The slight shift of this peak to higher temperatures suggests an interaction between the Ir and Ba species, where a more stable Ir^0^ could be created by introducing Ba.

Furthermore, the overall H_2_ consumption of the catalyst decreases after the introduction of Ba. Ba can act as an oxidation inhibitor and stabilize the catalytically active Ir species, enhancing activity and durability [27,28]. Therefore, the decrease in H_2_ consumption (in the H_2_-TPR results) suggested the presence of less IrO_2_ and more Ir^0^ in IrBa0.2/ZSM-5(27) than in Ir/ZSM-5(27).

### 3.8. X-ray Photoelectron Spectroscopy (XPS) of as-Made Catalysts

**Figure 10:**
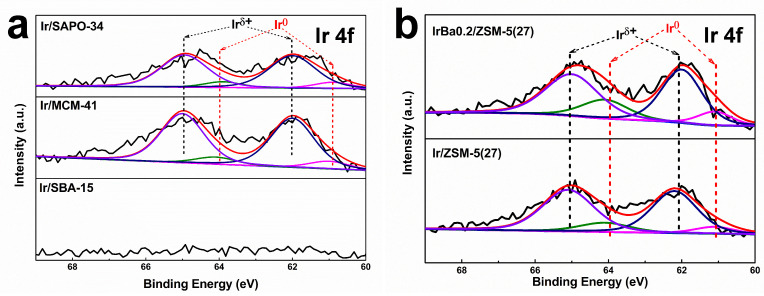
The XPS spectrum of Ir 4f for Ir/SAPO-34, Ir/MCM-41, and Ir/SBA-15 (**a**). The XPS spectrum of Ir 4f for Ir/ZSM-5(27) and IrBa0.2/ZSM-5(27) (**b**).

The Ir 4f signals of the Ir loaded onto different carriers are shown in Figure 10a. The binding energies (BEs) at 61.1 and 64.1 eV were attributed to Ir^0^, and those at 62.1 and 65.1 eV to electron-deficient iridium species (Ir^δ+^) [46,47], indicating that Ir^0^ and oxidized Ir^δ+^ coexisted in samples. Peak fitting and area analysis revealed contributions of 12.8% Ir^0^ and 87.2% Ir^δ+^ for Ir/SAPO-34 and 12.1% Ir^0^ and 87.9% Ir^δ+^ for Ir/MCM-41 (Table 3). Notably, the peak intensity of Ir/SBA-15 was very weak. This result indicates that most of the Ir species were located in the inner channels of SBA-15, as confirmed by the TEM image of Ir/SBA-15 (Figure 7g).

The Ir 4f spectra of Ir/ZSM-5(27) and IrBa0.2/ZSM-5(27) were also recorded, and the results are shown in Figure 10b. The proportion of Ir^0^ in Ir/ZSM-5(27) was 15.8%, which was higher than that in Ir/SAPO-34 and Ir/MCM-41. Because Ir^0^ sites are the active sites for the CO-SCR reaction, a higher proportion of Ir^0^ in Ir/ZSM-5(27) improves the NO*_x_* removal efficiency. Furthermore, the proportion of Ir^0^ increased to 23.8% after Ba modification, indicating that Ba modification was beneficial for the formation of metallic species (Ir^0^) in an oxidizing atmosphere. This result is consistent with the H_2_-TPR results, which also indicated a higher Ir^0^ distribution on the surface of IrBa0.2/ZSM-5(27) than on the other catalysts.

Considering that the Ir species in the metallic Ir^0^ form act as active sites in the CO-SCR reaction, Ba enhances the oxidation resistance of Ir^0^, which enables Ir to maintain its active metallic state, thereby significantly improving the catalytic activity and cyclic stability of Ir-based catalysts in the CO-SCR reaction when O_2_ is present.

## 4. Conclusions

The catalytic activity and cyclic stability of the bimetallic Ba-Ir catalyst supported on the ZSM-5 zeolite were studied for possible industrial applications in the selective catalytic reduction of NO by CO in oxygen-enriched environments. The catalyst carrier can influence the form of the Ir species and result in differences in the proportion of metallic Ir^0^ in these catalysts. The metallic Ir^0^ species were verified to be the active sites for CO-SCR, and H_2_ pretreatment resulted in more Ir^0^ species serving as active sites for CO-SCR. The activity of the Ir/ZSM-5 catalyst decreased by 17.6% in the three cyclic tests because of the oxidation of Ir^0^, which was recovered by H_2_ regeneration. Ba acts as an oxidation inhibitor and stabilizes the catalytically active Ir^0^ species, tailoring the Ir valence states on the catalysts and resulting in enhanced activity and high durability in the CO-SCR reaction in oxygen-enriched environments. NO*_x_* conversion was further improved to 77.1% by the addition of NH_3_ (NH_3_-CO-SCR). Therefore, the proposed catalyst is a promising alternative for stationary industrial applications in oxygen-enriched environments, such as flue gas treatment systems for steel plants, biomass-fired generators, and waste incinerators.

## Figures and Tables

**Table 1:** The NO conversion of different catalysts in the presence of O_2_.

Catalysts	Reaction Conditions	Ir Loading Amount (%)	NO Conversion (%)	References
IrRu/Al_2_O_3_	50 ppm NO, 7000 ppm CO, 5% O_2_	1.9%	88%	[12]
BaIrRu/Al_2_O_3_	50 ppm NO, 7000 ppm CO, 5% O_2_	1.9%	90%	[34]
IrW-WO_3_/KIT-6	1000 ppm NO, 4000 ppm CO, 5% O_2_	1.0%	<60%	[5]
Ir/WO_3_	500 ppm NO, 3000 ppm CO, 5% O_2_, 2 ppm SO_2_, 1% H_2_O	5.0%	25%	[31]
Ir/Al_2_O_3_	500 ppm NO, 1000 ppm CO, 300 ppm C_3_H_6_, 0.5% O_2_, 5% H_2_O	1.0%	nearly 80%	[35]
IrBa0.2/ZSM-5	600 ppm NO, 3000 ppm CO, 5% O_2_	0.5%	68%	This work

**Table 2:** The texture properties of as-made catalysts.

Sample	Specific Surface Area (m^2^/g)		Pore Volume (cm^3^/g)	
	S_micro_	S_total_	V_micro_	V_total_
Ir/SAPO-34	658.2	679.3	0.26	0.29
Ir/MCM-41	-	535.9	-	0.39
Ir/SBA-15	-	372.2	-	0.91
Ir/ZSM-5(27)	362.1	415.9	0.15	0.25
IrBa0.1/ZSM-5(27)	197.4	251.8	0.10	0.20
IrBa0.2/ZSM-5(27)	291.0	370.4	0.14	0.27
IrBa0.4/ZSM-5(27)	234.8	301.6	0.12	0.24
IrBa1.0/ZSM-5(27)	247.1	323.9	0.12	0.27

**Table 3:** Surface element states on different catalysts.

Samples	Ir^0^/(Ir^0^ + Ir^δ+^)
Ir/SAPO-34	12.8%
Ir/MCM-41	12.1%
Ir/SBA-15	-
Ir/ZSM-5(27)	15.8%
IrBa0.2/ZSM-5(27)	23.8%

## Data Availability

Data are contained within the article.
